# Fire Resistance of Geopolymer Foams Layered on Polystyrene Boards

**DOI:** 10.3390/polym14101945

**Published:** 2022-05-11

**Authors:** Van Su Le, Van Vu Nguyen, Artem Sharko, Roberto Ercoli, Thang Xiem Nguyen, Doan Hung Tran, Piotr Łoś, Katarzyna Ewa Buczkowska, Stanisław Mitura, Tomáš Špirek, Petr Louda

**Affiliations:** 1 Department of Material Science, Faculty of Mechanical Engineering, Technical University of Liberec, Studentska 2, 461 17 Liberec, Czech Republic; nguyen.van.vu@tul.cz (V.V.N.); artem.sharko@tul.cz (A.S.); piotr.los@tul.cz (P.Ł.); katarzyna.ewa.buczkowska@tul.cz (K.E.B.); stanislaw.mitura@tul.cz (S.M.); tomas.spirek@gec-gec.cz (T.Š.); petr.louda@tul.cz (P.L.); 2 Department of Pure and Applied Sciences, University of Urbino, Via Ca’ Le Suore 2/4, 61029 Urbino, Italy; r.ercoli@campus.uniurb.it; 3 Faculty of Civil Engineering, Nha Trang University, Nguyen Dinh Chieu 2, Nha Trang 650000, Vietnam; xiemnt@ntu.edu.vn; 4 Faculty of Mechanical Engineering, Nha Trang University, Nguyen Dinh Chieu 2, Nha Trang 650000, Vietnam; hung.tulcz@yahoo.com; 5 Department of Materials Technology and Production Systems, Faculty of Mechanical Engineering, Lodz University of Technology, Stefanowskiego 1/15, 90-537 Lodz, Poland; 6 Accademia Kaliska im. Prezydenta Stanislawa Wojciechowskiego w Kaliszu, Nowy Świat 4, 62-800 Kalisz, Poland

**Keywords:** geopolymer foams, metakaolin, silica sand, silica fume, chopped basalt fibers, aluminum powder, spray method, low thermal conductivity, high fire resistance

## Abstract

Geopolymer foams are excellent materials in terms of mechanical loads and fire resistance applications. This study investigated the foaming process of geopolymers and foam stability, with a focus on the fire resistance performance when using polystyrene as the base layer. The main purpose is to define the influence of porosity on the physical properties and consequently to find applications and effectiveness of geopolymers. In this study, lightweight materials are obtained through a process called geopolymerization. Foaming was done by adding aluminum powder at the end of the geopolymer mortar preparation. The interaction between the aluminum powder and the alkaline solution (used for the binder during the mixing process) at room temperature is reactive enough to develop hydrogen-rich bubbles that increase the viscosity and promote the consolidation of geopolymers. The basic principle of thermodynamic reactions responsible for the formation of foams is characterized by hydrogen-rich gas generation, which is then trapped in the molecular structure of geopolymers. The geopolymer foams in this study are highly porous and robust materials. Moreover, the porosity distribution is very homogeneous. Experimental assessments were performed on four specimens to determine the density, porosity, mechanical strength, and thermal conductivity. The results showed that our geopolymer foams layered on polystyrene boards (with optimal thickness) have the highest fire resistance performance among others. This combination could withstand temperatures of up to 800 °C for more than 15 min without the temperature rising on the insulated side. Results of the best-performing geopolymer foam underline the technical characteristics of the material, with an average apparent density of 1 g/cm^3^, a volume porosity of 55%, a thermal conductivity of 0.25 W/mK, and excellent fire resistance.

## 1. Introduction

In the second half of the 20th century, J. Davidovich was the first to introduce the concept of geopolymers [1], as synthetic three-dimensional inorganic polymers formed by polycondensation of aluminosilicate materials (rock-forming minerals, generally metakaolin) in a strongly alkaline environment [2]. The geopolymerization process involves a fast chemical reaction under alkaline conditions, resulting in 3D polymeric chains, constituted by Si-O and Al-O bonds [3]. The general mechanism for the alkali activation of silico-aluminate materials can be resumed into three main phases [4]: (i) dissolution, (ii) condensation, and (iii) stabilization.

Over the last decades, the development of geopolymers has become prominent and relevant research because of the great potential to be an alternative to the Portland-cement-based binders. The introduction of geopolymers not only meets the requirements for mechanical performance but it represents a new valid green construction material. Indeed, OPC manufacturing demands large amounts of energy and releases a significant amount of greenhouse emissions, estimating around 7% of the CO_2_ generated globally [5]. The experimental and innovative composite very often shows new properties, and it is, therefore, necessary to accurately characterize the obtained materials. A specific variant is the so-called geopolymer foam, which is also an excellent alternative to mineral wool, glass, and foam concrete. One of the greatest advantages of geopolymer foams is their low density and extremely low thermal conductivity [6,7].

Foaming methods to reduce the geopolymer density have been investigated, as lightweight geopolymers are continuously being studied to improve the insulating properties [8]. The foaming process is achieved by combining the binder, composed of aluminosilicate, and an aqueous alkaline solution with a metal powder (aluminum, or other metals). The result is that a hydrogen-rich gas is generated and released during metal oxidation [9,10,11,12,13,14], leading to the formation of a porous structure that is cured [15,16,17,18] and stabilized with solid particles [19]. The stabilization facilitates the dispersion of the hydrogen gas in tiny bubbles and increases the strength of the thin films between them [20,21,22]. Foams resemble condensed emulsions in their structure, but the dispersed phase is a gas, not a liquid [23,24]. Definitively, the porous structure of geopolymer foam is a modern technical achievement widely used to produce low-density geopolymers, which ensures a porosity pattern [25,26,27,28].

In this study, the physical properties of four different geopolymer foams (density, porosity, compressive–flexural strengths, and thermal conductivity) have been investigated. Moreover, the effect of high temperatures on the geopolymer coatings has been evaluated to determine fire resistance. The main constituent of geopolymers consists of metakaolin activated in an aqueous alkaline solution (pH 11), chopped basalt fibers, silica sand, silica fume, and aluminum powder used for the foaming process. Usually, depending on the starting materials and manufacturing processes, geopolymer foams are good thermal insulators, and fire-resistant coatings can be manufactured from them. Additionally, the dielectric resistance properties of the foamed materials are improved to protect them from the influence of the environment [29,30,31,32,33].

Following these assumptions, the final aim of the research is to achieve materials with high-performing properties, especially thermal and fire resistance, keeping good mechanical properties. This standard is achievable only via a controlled foaming process that leads to the formation of well-distributed pores. In this way, the structure of geopolymers has been wholly preserved after experimental tests carried out on polystyrene boards to determine the fire resistance.

The novelty of the study concerns the building sector with the potential use of the aforementioned geopolymer foams as cladding materials with insulating and refractory properties. The technical characteristics of these innovative materials make it possible to mitigate problems such as fire propagation. An example is the Grenfell tower fire on 14 June 2017 [34,35,36,37,38,39], where a fire started by a fridge malfunctioning on the fourth floor. The fire spread rapidly up the building’s exterior in less than 15 min, bringing fire and smoke to all the other residential floors. This was due to the building cladding and the external insulation since the air gap between them enabled the stack effect. Therefore, this effect could be mitigated by filling this cavity using the foamed materials, lowering the risk of fire spreading and increasing the time delay of propagation from the fire source to the other environments.

## 2. Materials and Methods

### 2.1. Raw Materials Employed for Synthesizing Geopolymer Foams (GFs)

**Figure 1:**
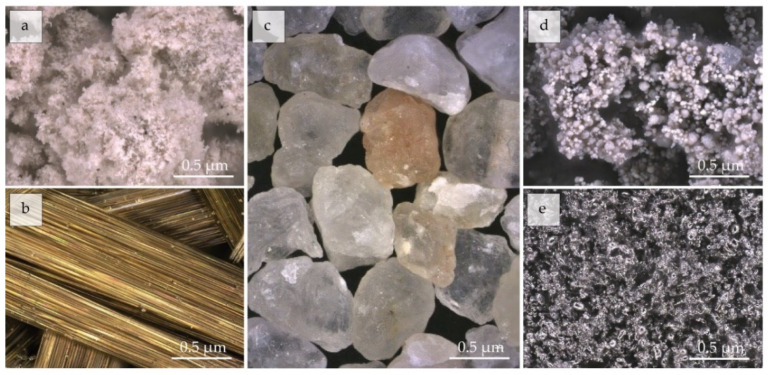
(**a**) Metakaolin, (**b**) chopped basalt fibers, (**c**) silica sand, (**d**) silica fume, and (**e**) Al.

The parameters through which raw materials (Figure 1) affect the resulting geopolymer foams are (i) chemical composition, (ii) mineralogy, and (iii) granulometry. These three fundamentals are responsible for the reactivity of the alkaline activator and the formation of the optimal pore structure of the cellular composite.

The sequence of processes that lead to the production of geopolymer foams is given by the annealing of kaolinite clay, which allows the destruction and dehydration of clay minerals to form metakaolin (MK) (kaolinite roasted product). This process is followed by the bond breaks of the metakaolin structure via the alkaline activator (A). The dissolution of aluminosilicate materials and the transition of silicate and aluminate anions into the liquid phase is the stage that represents the kinetics of the hardening process of geopolymer materials. In an alkaline medium, dissolution of the aluminosilicates occurs with the formation of aluminate and low-polymer silicate anions. This process ends with the formation of active monomeric and low-polymer ions in solution, whereas the hardening mechanism of the geopolymer ends with the re-condensation of low polymer and aluminosilicate ions.

In the experimental methods, the physical properties of the geopolymer foams are determined by the chemical composition, which depends on the nature of the starting materials and the hardening process parameters. Therefore, specific raw materials were employed for the production of the geopolymer foams.

The industrially commercial binder “Baucis lk” used for this research was supplied by České Lupkové Závody, a.s. (Nové Strašecí, Czech Republic) and is a two-component aluminosilicate binder based on metakaolin (MK) activated by an alkaline solution of potassium hydroxide (A). The metakaolin (MK) was used as precursor material, obtained by calcining kaolinite at temperatures 500–800 °C (the more active the aluminosilicate precursor material, the higher the rate of physical strength). Unlike mineral products, metakaolin is characterized by composition, morphology, and particle size homogeneity. Therefore, it is often used as a standard for studying the formation of geopolymers.

Chopped basalt fibers (CBFs) were added to the mixture to improve the mechanical properties of geopolymers. The stabilization process using the fibers is widely demonstrated, increasing the viscosity of the paste. Basaltex, a.s., (Šumperk, Czech Republic) provided CBFs, characterized by a fiber length of 3.2 mm, a diameter of 13 µm, a density of 2.67 g/cm^3^, and thermal conductivity of 0.031/0.038 W/mK.

Silica sand (SA) was employed as a coarse material to structure the geopolymer. In addition to being a good heat conductor, the mechanical properties of geopolymers are improved. Sklopisek Strelec, a.s., (Hrdonovice, Czech Republic) supplied the silica sand characterized by a mean range particle size of 0.3–0.8 mm and a density of 2.65 g/cm^3^.

The improvement of fire resistance properties of geopolymers is ensured by fire-retardant additives (silica fume and aluminum powders). Such additives increase the autoignition temperature and reduce the self-extinguishing time and weight loss during combustion. At the same time, introducing additives as fire-retardant systems requires a high filler content in the polymer matrix, having a high density but low mechanical properties. The silica fume (SF) (produced by Kema Morava—sanační centrum a.s., Republic of Slovenia) contained 90 wt.% of SiO_2_, with an average grain size of 1 mm. The aluminum powder (Al) was supplied by Pkchemie, Inc. (Třebíč, Czech Republic) with a mean particle size of 51.47 µm. By interacting aluminum powder with the aqueous alkaline solution used for the activation process, hydrogen-rich gas mixtures were generated and trapped since the gas and polymer phases existed separately.

The chemical compositions were determined using X-ray fluorescence (BRUKER S8 Tiger instrument, BRUKER, Karlsruhe, Germany). Results are summarized in Table 1.

### 2.2. Methods for the Synthesis of Geopolymer Foams (GFs)

The predetermined ratios of the GFs are given in Table 2. The mixing procedure of the geopolymer mortar is as follows: (i) the metakaolin (MK) was mixed with an alkaline solution of potassium hydroxide (A) (KOH, pH = 11) for five minutes; (ii) the chopped basalt fibers (CBFs), silica sand (SA), and silica fume (SF) were added and stirred for another five minutes, (iii) and finally, aluminum powder (Al) was mixed for one minute.

Hardening treatments of the experimental products were carried out under heat drying conditions at room temperature. All the GF specimens were cured in specific molds for 28 days to test their effective physical properties. The curing rate can be studied by considering the kinetic transformations during the hardening process.

### 2.3. Characterization Methods for GFs

The experimental study examined the pore structure, mechanical properties, thermal conductivity, and fire resistance of the geopolymer foams.

The pore size distributions of the GFs were determined using an AutoPore IV 9510 mercury intrusion porosimeter, which operates at pressures ranging from 0.01 to 414 MPa. The samples were tested on 40 × 40 × 10 mm^3^ plates. For further details, reference is made to work [40].

Compression and bending tests were conducted using an Instron (Model 4202) Universal Testing Machine with a load cell of 10 kN and a crosshead speed of 2.5 mm/min at ambient temperature. Specifically, the compression strengths were measured on cubic specimens of 40 mm^3^, and the three-point bending strength on specimens of 40 × 40 × 160 mm^3^. For each series, three samples were used to calculate the mean compressive and flexural strengths. Tests were conducted under CSN EN 1015-11 [41].

The device model HFM436 Lambda (Netzch, a.s., Selb, Germany) [42] was used to analyze the thermal conductivity. Note that the sample dimensions required for this measurement are 300 × 300 × 50 mm^3^.

### 2.4. Methods for Fire Resistance Evaluation of GFs Layers on Polystyrene Boards

**Figure 2:**
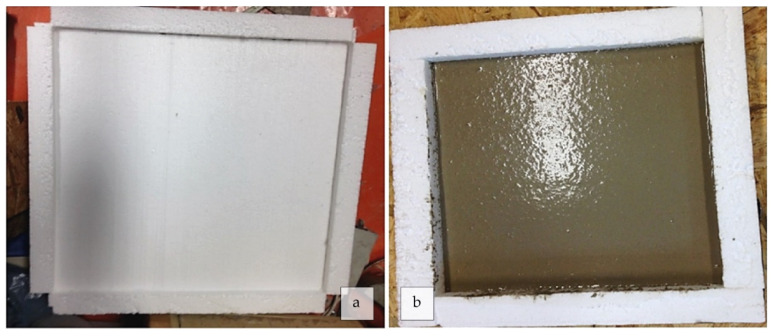
Preparation of three (**a**) polystyrene boards (500 × 500 mm^2^) for testing the fire resistance: (**b**) the GF (300 × 300 mm^2^) was coated on them with different thicknesses (20, 15, 10 mm).

Fire resistance tests of geopolymer foams were conducted through specific thicknesses of the geopolymer foam layer on three polystyrene boards. The layer was applied as a treated surface to increase the fire resistance of the polystyrene: three specimens with 20-, 15-, and 10-mm thick coatings (Figure 2). According to Table 2, (Section 2.2), the three specimens were prepared following the S20 composition. The specimens were cured on the boards (500 × 500 mm^2^) for 28 days at RT before testing. After the hardening process, each board was mounted in a furnace and the fire exposure area (300 × 300 mm^2^) gradually heated up to 800 °C.

**Figure 3:**
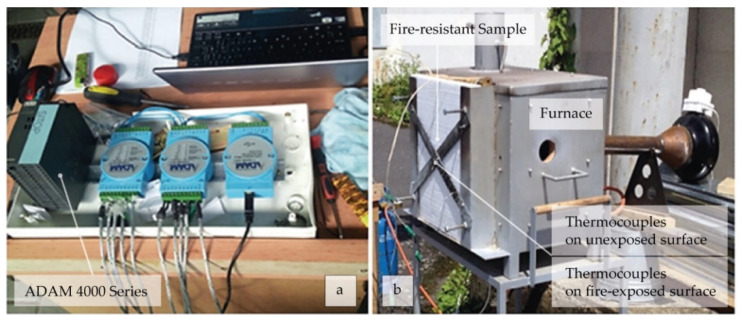
Equipment setup for monitoring the temperature variations: (**a**) ADAM 4000 Series and (**b**) internal (K5–K9) and external thermocouples (K1–K8).

The equipment as illustrated in Figure 3 was used to test the fire resistance according to CSN EN 13381-3 [43] by the technical committee of the Technical University of Liberec. The test furnace was heated with natural gas and the heating rate was controlled according to ISO 834-11:2014 standard [44]. In addition, the deformation of the steel plates and, e.g., warping defects of polystyrene were used as indicators for the fire resistance performance of the samples. The temperatures of the unexposed and exposed surfaces of the samples were measured using in-built thermocouples connected through the ADAM 4000 series system. Thermocouples K5 and K9 were located on the inner side of the furnace while thermocouples K1 and K8 were on the outer side.

## 3. Results and Discussion

### 3.1. Densities and Porosities of Geopolymer Foams

As previously explained, during the experimental studies, the foaming process of geopolymers aimed to create a cellular structure of air or other gaseous substances within the material. The structure was achieved using chemical blowing agents, specifically aluminum powder, that release gaseous products due to their chemical decomposition. The phenomenon of structure formation and foam flow during the consolidation process is a hydrodynamic process associated with blocking gas–mixture interaction.

The introduction of the gas phase into the geopolymer causes a sharp change in its physical characteristics. For example, the thermo-mechanical properties of the materials depend on it, and with the increase of the temperature within the geopolymer, the heat capacity of the gas increases within the pores. Specifically, the main morphological parameter of geopolymer foams is the apparent density value, which expresses the relative content of solid and gaseous phases in the geopolymer.

The porosimetry parameters are shown in Table 3. Increasing the filler content, the density of S22, S21, and S20 increases (compared to S19). Meanwhile, the porosities of S22, S21, and S20 (55.7%, 54.1%, 53.2%) decrease concerning S19 (56.9%). For geopolymer composites, density and percent porosity are negatively correlated [45].

When studying the properties of GFs, it is necessary to know the shape of the cells and their volume distribution since the cell density, shape, and geometry affect the strength of the final product. Definitively, the cell size distribution function is the most significant characteristic of the structure of polymer foams.

**Figure 4:**
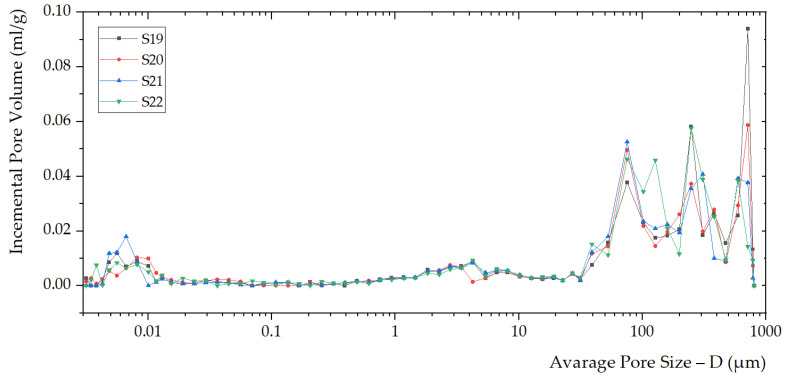
Porosity distribution within the foamed geopolymer foams S19, S20, S21, and S22 was identified through the average pore size (D) versus incremental pore volume.

**Figure 5:**
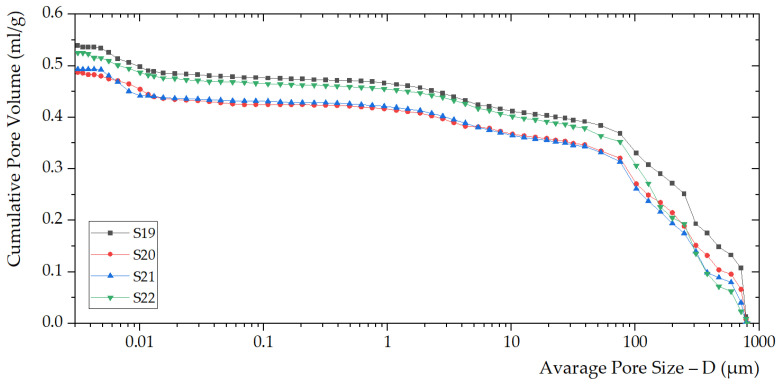
Curves of S19, S20, S21, and S22 illustrate the average pore size (D) versus cumulative pore volume.

Investigations on the foaming process kinetics require modern fracture mechanics concepts. The mechanism of instability observed during the foaming process is based on fluctuations in the bubble flow rate due to the geometric inhomogeneity of the pores. Relatively rapid foaming with large volumes is observed during pressure drops. Then, with a density increase, the sizes of the cellular structures decrease. In this case, the stabilization of the geopolymer structure occurs (Figure 4 and Figure 5).

The volume of the foam is useful for studying the structure parameters and their influence to obtain a low-density geopolymer. More in-depth, the physical properties of GFs strongly depend on the foam volume. At the same time, it is challenging to determine the volume accurately, and sometimes, it is not possible because the density depends on the aeration duration, pressure of compressed air from the foam source, and temperature variation of the system.

### 3.2. Mechanical Properties of GFs

**Figure 6:**
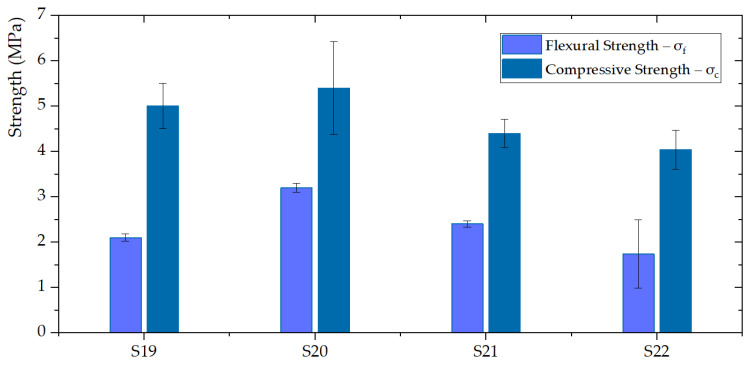
Compressive and flexural strengths of the GFs: S20 has been identified as the best performing geopolymer in terms of mechanical parameters.

The mechanical properties (compressive (σc) and flexural (σf) strengths) of GFs are presented in Figure 6. The flexural and compressive strengths of S20 (3.2 MPa and 5.4 MPa) increased compared to S19 (2.1 MPa and 5 MPa). On the other hand, the compression strength of S21 and S22 are 12% and 19.2% smaller than S19. The reason for this slight decrease is the differential amount in the content of silica fume. The structure of the geopolymer is affected by prominent changes when the silica fume is added, creating additional voids.

### 3.3. Thermal Conductivity of GFs

**Figure 7:**
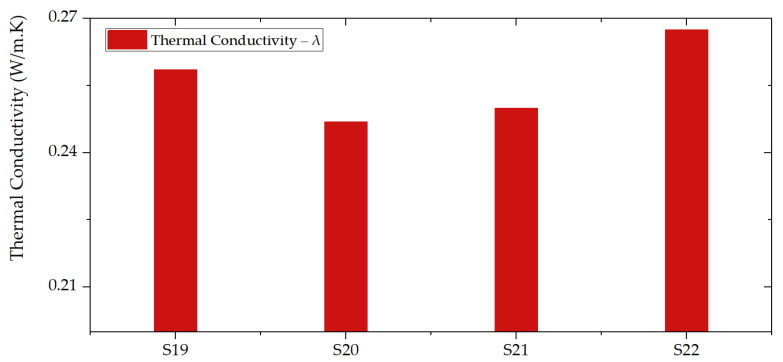
Thermal conductivity of GFs: S20 has the lowest thermal conductivity among the synthetized geopolymers.

Thermal activation is always required during the synthesis of geopolymer foams. Temperature significantly accelerates the initial process of GFs formation and thus has an essential effect on curing the process, especially in the initial time of the reactions. The excellent resistance of geopolymers to temperature changes makes them suitable for working in unfavorable conditions. The thermal conductivity (λ) of GFs is illustrated in Figure 7. Samples containing 20 wt.% of silica fume have greater λ than the samples containing less amount (5% and 10 wt.%). Sample S22 presents the highest λ (0.27 W/mK). On the contrary, sample S20 is the one with the lowest λ (0.25 W/mK). S20 and S21 with 5 wt.% and 10 wt.% of silica fume have lower λ than S19 without silica fume.

### 3.4. Fire Resistance of GFs Layers Sprayed on Polystyrene Boards

**Figure 8:**
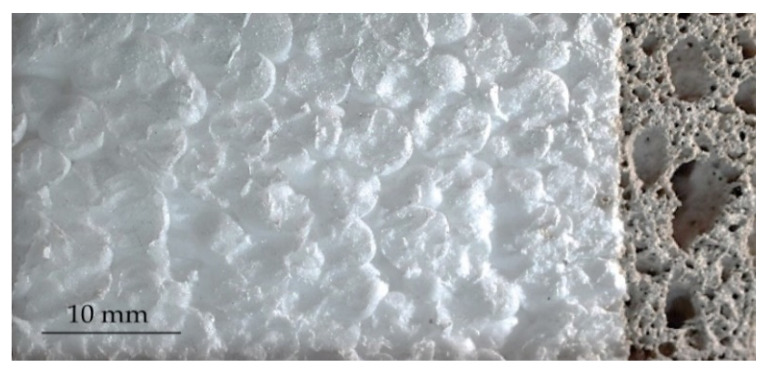
Section of the polystyrene board with a layer of S20. Three experimental tests were conducted with specimens 20, 15, and 10 mm thick.

Geopolymer S20 was recognized as the material with the best performance in terms of physical properties. Thus, only the fire resistances on polystyrene boards coated with the geopolymer foam S20 (Figure 8) were tested making the process simple and economic. The fire resistance tests were performed through specimens of different thicknesses (20, 15, and 10 mm) covering the polystyrene board as a treated surface.

**Figure 9:**
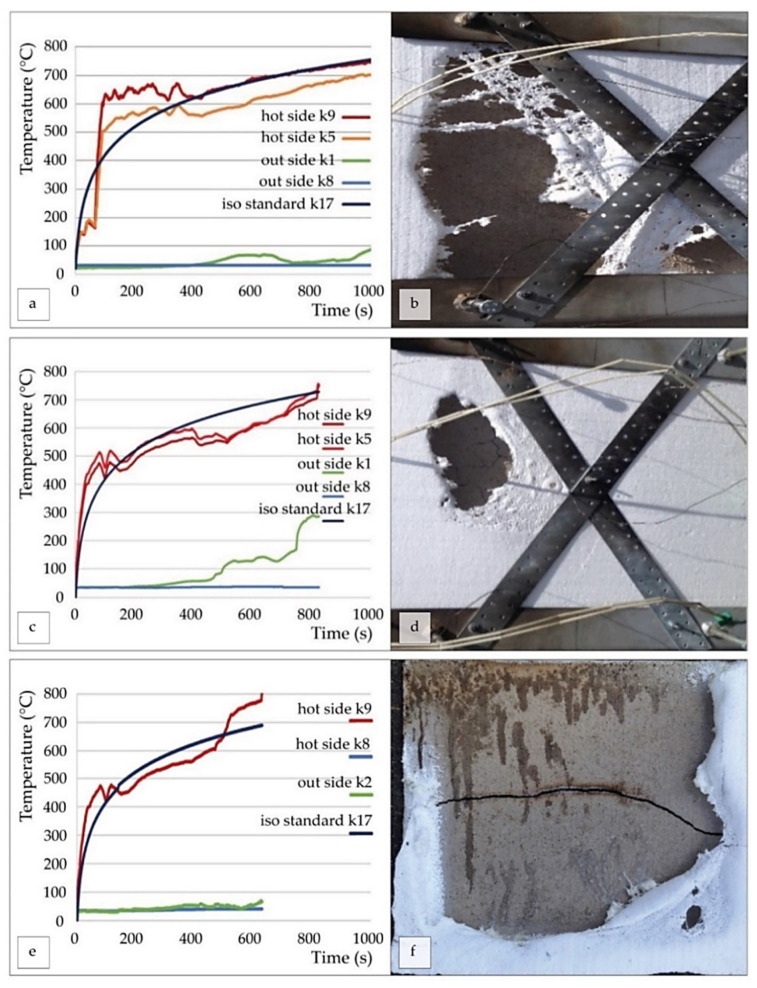
Fire resistance curves (**a**,**c**,**e**) and the outer surface of S20 (20, 15, 10 mm thick) after testing (**b**,**d**,**f**); K5, K9 and K1, K8 are the inner and outer thermocouples; K17 is the standard curve.

The temperature variations during all the tests are shown in Figure 9. The time interval of the fire resistance of treated polystyrene boards with GF layer was 1000 s for sample 1 (20 mm thick; Figure 9a,b), 800 s for sample 2 (15 mm thick; Figure 9c,d), and 600 s for sample 3 (10 mm thick; Figure 9e,f). The untreated polystyrene board in the same testing conditions was destroyed instantaneously.

The stepwise temperature increase can explain the thermomechanical properties of the material. Elastic deformation of the samples occurs in the initial phase of heating up to 200 °C. The differential gas pressure inside and outside the porous cells changes with the increase in temperature and the rigidity of the polymer matrix. Further, the pores expand due to heating and increase viscosity as the temperature rises above 200 °C. The stability of the chemical bonds occurs at temperatures above 600 °C. With a further increase in temperature, the thermal expansion is compensated by the pressure inside the pores stabilizing the structure.

## 4. Conclusions

A mechanism for producing metakaolin-based geopolymer foams through the use of fillers such as chopped basalt fibers, silica sand, silica fume, and aluminum powder has been proposed. Based on the experimental tests carried out, it has been proven that the foaming process by the aluminum powder decreases the thermal conductivity with the direct consequence of using these low-density geopolymer composites as fire resistance coatings material. Stabilization of the porous suspension during the foaming process occurs by adding chopped basalt fibers to the geopolymer mortar: an increase in the viscosity of the paste happens, which leads to a decrease in pore collapse.

Investigations on the apparent density, porosity distribution, mechanical strength, and fire resistance properties have confirmed the potentiality of the geopolymer foam as a brand new technology for promoting the use as fire-retardant materials in the building sector. The optimal strength and thermal conductivity combination have been developed by using the GF named S20 (49.01 wt.% SiO_2_, 11.39 wt.% Al_2_O_3_, 6.35 wt.% CaO, 0.94 wt.% MgO, 0.47 wt.% TiO_2_, 0.45 wt.% Fe_2_O_3_, 0.3 wt.% K_2_O, 0.17 wt.% SO_3_, 0.11 wt.% MnO, 0.12 wt.% Na_2_O, 0.003 wt.% P_2_O_5_). The porous geopolymer has a density of 53.2%, a total pore volume of 0.487 mL/g, flexural strength of 3.2 ± 0.1 MPa, compressive strength of 5.4 ± 1.02 MPa, and low thermal conductivity (0.25 W/mK), and 20 mm of this material coated on a polystyrene board can resist 1000 s against temperatures up to 800 °C.

Definitively, the distinctive feature of this study is the use of S20 as fire-resistant material for the construction industry. As highlighted in the experimental results, the geopolymer foam applied as a coating on the polystyrene boards increases the fire resistance of the material. If this concept is applied to the building sectors, it shows important requirements to reduce the risk of fire propagation. Moreover, from an economical point of view, the possibility of purchasing the mixture components directly at the enterprises of the Czech Republic significantly reduces the cost of production.

## Figures and Tables

**Table 1:** Chemical compositions of main constituents of the GFs (wt.%).

Constituents	SiO_2_	Al_2_O_3_	CaO	MgO	TiO_2_	Fe_2_O_3_	K_2_O	SO_3_	MnO	Na_2_O	P_2_O_5_	LOI
Metakaolin (MK)	44.5	28.9	17.6	2.23	1.31	0.82	0.75	0.46	0.28	0.25	-	2.56
Chopped Basalt Fibers (CBFs)	33.6	14.4	26.1	8.26	1.98	6.61	1.21	0.29	0.76	1.38	0.14	2.05
Silica Sand (SA)	99.4	-	-	-	-	0.05	-	-	-	-	-	-
Silica Fume (SF)	83.9	1.54	1.07	1.5	-	1.07	1.98	0.917	-	0.367	-	4.72
Aluminum Powder (Al)	0.07	99.4	-	-	-	0.11	-	-	-	-	-	-

**Table 2:** The ratio of the GFs composition (binder—B, and fillers—Fs) is referred to MK.

GFs	Binder (B)		Fillers (Fs)			
	Metakaolin (MK)	Activator (A)	Chopped Basalt Fibers (CBFs)	Silica Sand (SA)	Silica Fume (SF)	Aluminum Powder (Al)
**S19**	1	0.9	0.07	1	0	0.05
**S20**					0.05	
**S21**					0.1	
**S22**					0.2	

**Table 3:** Summary of the porosimetry results.

Parameters	Unit	S19	S20	S21	S22
Total Pore Volume	mL/g	0.539	0.487	0.493	0.525
Total Pore Area	m^2^/g	35.6	32.3	36.8	34.5
Median Pore Diameter (volume)	μm	183.57	122.49	103.62	119.06
Median Pore Diameter (area)	μm	0.0056	0.0064	0.0059	0.0054
Average Pore Size (4 V/A)	μm	0.0605	0.0604	0.0537	0.0608
Density at 0.0015 MPa	g/mL	1.0550	1.0914	1.0974	1.0606
Apparent (Skeletal) Density at 413.3967 MPa	g/mL	2.4450	2.3302	2.3918	2.3921
Porosity	%	56.9	53.2	54.1	55.7

## Data Availability

The data presented in this study are available on request from the corresponding author.
